# What Are the Effective Components of Group-Based Treatment Programs For Smoking Cessation? A Systematic Review and Meta-Analysis

**DOI:** 10.1093/ntr/ntad068

**Published:** 2023-04-27

**Authors:** Amanual Getnet Mersha, Jamie Bryant, Tabassum Rahman, Romany McGuffog, Raglan Maddox, Michelle Kennedy

**Affiliations:** College of Health Medicine and Wellbeing, University of Newcastle, Callaghan, NSW, Australia; Hunter Medical Research Institute, New Lambton Heights, NSW, Australia; College of Health Medicine and Wellbeing, University of Newcastle, Callaghan, NSW, Australia; Hunter Medical Research Institute, New Lambton Heights, NSW, Australia; Centre for Epidemiology and Biostatistics, University of Melbourne, VIC, Australia; College of Health Medicine and Wellbeing, University of Newcastle, Callaghan, NSW, Australia; National Centre for Epidemiology and Public Health, Australian National University, Canberra, ACT, Australia; College of Health Medicine and Wellbeing, University of Newcastle, Callaghan, NSW, Australia; Hunter Medical Research Institute, New Lambton Heights, NSW, Australia

## Abstract

**Introduction:**

There is significant variation in the format and delivery of group-based smoking cessation programs. To guide research and healthcare program implementation, it is important to understand the active components of interventions.

**Aims and Methods:**

This review aimed to (1) identify behavior change techniques (BCTs) used in effective group-based smoking cessation interventions, (2) determine the effectiveness of group-based smoking cessation interventions on smoking cessation at 6-month follow-up, and (3) identify the behavior change techniques (BCTs) related to effective group-based smoking cessation. The following databases were searched in January 2000 and March 2022: MEDLINE, EMBASE, CINAHL, PsycINFO, The Cochrane Library, and Web of Science. BCTs used in each study were extracted using the BCT Taxonomy. Studies that included identified BCTs were computed, and meta-analyses were conducted to evaluate smoking cessation at 6-month follow-up.

**Results:**

A total of 28 BCTs were identified from 19 randomized controlled trials. Studies included an average of 5.42 ± 2.0 BCTs. The most frequent BCTs were “information about health consequences” and “problem solving.” The pooled 6-month smoking cessation was higher in the group-based intervention group (OR = 1.75, 95% CI = 1.12 to 2.72, *p* <.001). Inclusion of the following four BCTs: “Problem solving,” “Information about health Consequences,” “Information about social and environmental consequences,” and “Reward (outcome)” were found to be significantly associated with increased rate of 6-month smoking cessation.

**Conclusions:**

Group-based smoking cessation interventions doubles the rate of smoking cessation at 6-month follow-up. Implementing group-based smoking cessation programs, that incorporate multiple BCTs, is recommended for an effective smoking cessation care.

**Implications:**

Group-based smoking cessation programs improves smoking cessation outcomes in clinical trials. There is a need to incorporate effective individual BCTs techniques to enhance smoking cessation treatment outcomes. A robust evaluation is required to assess the effectiveness of group-based cessation programs in real world settings. There is also a need to consider the differential effectiveness of group-based programs and BCT impacts on populations, for example, indigenous peoples.

## Introduction

Fueled by the Tobacco Industry,^1^ commercial tobacco smoking is a major global cause of premature death and disability that claims the lives of more than 8 million people every year.^2^ In 2017, it was estimated that more than 1.1 billion people continued to smoke worldwide.^3^ In response to the Tobacco Industry fueled global epidemic, the World Health Organization (WHO) developed an evidence-based treaty, the WHO Framework Convention on Tobacco Control.^4^ The WHO Framework Convention on Tobacco Control is developed to protect present and future generations from the devastating impacts of tobacco smoking. To this end, article 14 “Demand reduction measures concerning tobacco dependence and cessation” of the convention urges countries to design and implement effective programs to ­promote smoking cessation and curb the impact of tobacco products.^4^

Smoking cessation is considered one of the most effective and cost effective strategies to improve an individual’s health and wellbeing.^5^ Over the years, the effectiveness of a variety of smoking cessation interventions have been examined across the world.^3,6^ Interventions including brief physician advice, the provision of printed information resources, pharmacotherapies, text messaging, and internet-based treatment programs have demonstrated significant improvements in smoking cessation, including in systematic reviews and meta-analyses.^3,4,7^ A mainstay of smoking cessation support is the provision of behavioral counseling, which can be provided individually and in groups.^8^ There is high-quality evidence that behavioral counseling for people who smoke can assist in successfully quitting, with more intensive behavioral counseling more effective than brief counseling.^9^

Group-based smoking cessation interventions provide opportunities to learn behavioral techniques for smoking cessation, while also facilitating social support, a sense of belonging, social learning, and the sharing of knowledge, skills, and experiences.^10,11^ Group-based smoking programs are often provided over a period of several weeks,and include a clinician-led discussion on health impacts of smoking, benefits, and challenges of quitting. This typically will be followed by experience sharing by group members, and/or role-playing on how to deal with various smoking triggers or managing withdrawal symptoms.^11–13^ In 2017, a Cochrane review was conducted to evaluate the effectiveness of group-based smoking cessation interventions. The review reported improved rates of smoking cessation for participants in the group-based intervention arms compared to usual care (risk ratio [RR] = 2.60, 95% CI = 1.80 to 3.76), self-help programs (RR = 1.88, 95% CI = 1.52 to 2.33), and brief individual support (RR = 1.22, 95% CI = 1.03 to 1.43).^14^ However, there is significant variation in the format and delivery of individual and group-based smoking cessation programs. To guide research and healthcare program implementation, it is important to understand the individual components of cessation interventions that make them effective.

In 2013, a taxonomy of behavior change techniques (BCT) was proposed with the dual purpose of supporting theory-informed behavior change interventions and encouraging consensus in reporting of the underlying components of behavior change interventions.^15^ The behavior change taxonomy included a hierarchical list of 93 behavior change techniques, which are “observable, reproducible and simplified components of an intervention designed to modify causal processes that regulate behavior.”^15^ BCTs allow the components of interventions to be described in a replicable format and allow meta-analysis to explore *how* and *why* interventions work, rather than just if they work. The BCT taxonomy was used successfully to identify the components and evaluate the effectiveness of internet-based smoking cessation programs.^16,17^

To date, no systematic review has examined the active components of group-based smoking cessation interventions using a predefined theoretical framework to represent the interventions in a more clear and replicable manner.

### Aims

Consistent with the WHO Framework Convention on Tobacco Control, the aims of this systematic review and meta-analysis were to:

(1) identify the most frequently occurring BCTs used in effective group-based smoking cessation interventions.
(2) determine the effectiveness of group-based smoking cessation interventions on rates of smoking cessation at 6-month follow-up.
(3) identify the individual BCTs and BCTs domains related to effective group-based smoking cessation interventions at 6-month follow-up.

## Methods

### Protocol Registration

This review is registered on PROSPERO with registration number CRD42022318308 and can be accessed at https://www.crd.york.ac.uk/prospero/display_record.php?ID=CRD42022318308

### Search Strategy

This systematic review followed the Preferred Reporting Items for Systematic Reviews and Meta-Analyses (PRISMA) guideline.^18^ A literature search was conducted using six databases: MEDLINE, EMBASE, CINAHL, PsycINFO, The Cochrane Library, and Web of Science. All databases were searched from the date of inception to March 16, 2022, using a combination of keywords and phrases such as smoking, smoking cessation, cessation, smoke, cigarette, quitting, quitting smoking, group*, group therapy, group therap*, group therapy or cognitive therapy” (Supplementary Material 1). Additional searches were conducted on the National Institute of Health, Clinical Trials registry, and WHO International Clinical Trials Registry Platform. The reference lists of included articles and subject-specific journals, including Addiction, Nicotine and Tobacco Research, and Tobacco Control were also searched to identify publications.

### Inclusion and Exclusion Criteria

Studies were included if they were published in the English language between January 2000 to March 2022, were randomized controlled trials (RCTs) conducted with people who currently smoked and aimed to examine the effectiveness of a group-based smoking cessation program. For the purposes of this review, group-based smoking cessation programs were defined as any intervention where people who smoked met for scheduled meetings and received any form of intervention (eg, information, motivation, or cognitive behavioral therapy). Studies in which participants received group-based interventions in addition to any other active treatment programs (eg, self-help programs, individual counseling, or pharmacological interventions) were included. No exclusion was made based on the smoking cessation measures. There were no restrictions on study population, with studies that enrolled the general population, as well as those that enrolled specific subpopulations (eg, pregnant women, individuals of low socioeconomic status, specific ethnic groups, or disease conditions) included. Studies with a comparator arm of no intervention, placebo intervention, usual care, or active treatments as comparators were included.

### Record Screening

Citations were exported into covidence^19^ for initial screening. All retrieved records were independently screened by two reviewers (AM and MK) and assessed against the inclusion and exclusion criteria based on the information contained in the title and abstract. Full-text versions of papers initially determined as meeting the inclusion and exclusion criteria were the obtained and independently reviewed by two authors (AM and MK). Studies which met all inclusion criteria were retained for inclusion in the review. The reason for exclusion was recorded for studies that did not meet all inclusion criteria. Disagreements between reviewers were resolved through discussion.

### Data Extraction

A data extraction tool was developed to elicit key characteristics of included studies. For example, first author name, year of publication, main outcomes, study location (country), participant characteristics (age, gender, and race or ethnicity) , sample size, study design, implemented intervention details (components of comparators, intervention settings, frequency, duration, and modes of intervention delivery), the smoking cessation outcome assessments used and theories and treatment guidelines used to inform interventions. International associations, such as Consolidated Standards of Reporting Trials (CONSORT)^20^ and the UK Medical Research Council (MRC), recommend the use of frameworks to identify and report components of interventions.^21,22^ BCTs used in each intervention were, therefore, extracted using the BCT Taxonomy v1.^15^ The BCT Taxonomy v1 was developed through a series of Delphi exercises involving behavioral change experts and includes 93 BCTs in 16 domains. The description of each intervention was mapped to the domains and BCTs within the BCT Taxonomy independently by two reviewers who have experience in using the framework (AM and TR). Cohen’s kappa statistic was used to evaluate intercoder reliability. Interrater reliability checks on the identification of BCTs were conducted for each study, as well as the mean score across the included studies. A kappa score of values ≤0.20 was regarded as none to slight agreement, 0.21–0.40 as fair, 0.41–0.60 as moderate, 0.61–0.80 as substantial, and 0.81–1.00 as almost perfect agreement between coders.^23^ Disagreements were resolved through discussion and involving a third reviewer (MK) where necessary.

### Risk of Bias (Quality) Assessment

Two reviewers (AM and RMG) independently assessed the quality of studies using the Joanna Briggs Institute (JBI) Critical Appraisal tools for RCTs.^12^ The quality assessment tool evaluates various components of the methodology such as randomization, selection bias, attrition bias, bias arising from adherence, controlling for confounding factors, study power, and the strength of causality in the association between interventions and outcomes. Disagreements were resolved though discussion.

### Data Synthesis and Analysis

#### Aim 1

##### Identify the most frequently occurring BCTs used in effective group-based smoking cessation interventions.

Descriptive statistics were used to characterize included trials, such as the number and type of BCTs domain(s); and frequency, providers, and setting of the intervention groups. Effective interventions were defined as those that reported a statistically significant (*p* < .05) difference in smoking between the intervention and comparator groups (either self-reported or biochemically verified) at the final follow-up assessment.

#### Aim 2

##### Determine the effectiveness of group-based smoking cessation interventions on smoking cessation rates at 6-month follow-up.

Reporting 6- and/or 12-month abstinence rates are recommended by various smoking cessation measurement guidelines such as the Russell Standard^24^ and the Society for Research on Nicotine and Tobacco workgroup on abstinence measures used in trials of smoking cessation interventions.^25,26^ On the basis of this, studies that included BCTs and reported 6-month smoking cessation outcomes were computed for analysis using Stata software (V16, Stata Corp LP, College Station, TX). The number of individuals who were and were not abstinent at 6-month follow-up was recorded for each study. When a study reported both self-report and biochemical validated outcomes, we used the biochemical validated outcomes. Active comparator groups were used when there are more than two arms in the trial, that is, when there is a control, individual-based and group-based intervention comparisons, group-based intervention was compared with individual-based intervention. A pooled odds ratio with a 95% confidence interval was calculated using DerSimonian-Laird (DL) random-effects meta-analyse model. To determine the cause of heterogeneity, a subgroup analysis was conducted based on outcome measures (Continuous abstinence vs. point prevalence of abstinence at 6 months). Given the small number of studies that reported 6-month smoking cessation outcomes, 7-day point prevalence abstinence, and 30-day point prevalence abstinence were merged to compare with continuous abstinence.

#### Aim 3

##### Identify individual BCTs and BCT domains related to effective group-based smoking cessation interventions.

Meta-analysis was conducted if the domain/BCT appeared in at least two trials that assessed the rate of smoking cessation at 6-month follow-up. Forest plots were generated to show the overall effect sizes and 95% confidence intervals. Heterogeneity of estimates were assessed using the Higgins’ *I*^2^ statistical analysis test, a value of above 75% was considered substantial.^27^ Publication bias was evaluated using a funnel plot and Egger’s regression asymmetry test with a *p* value < .05 as a cutoff point.

## Results

### Search Results

**Figure 1.**
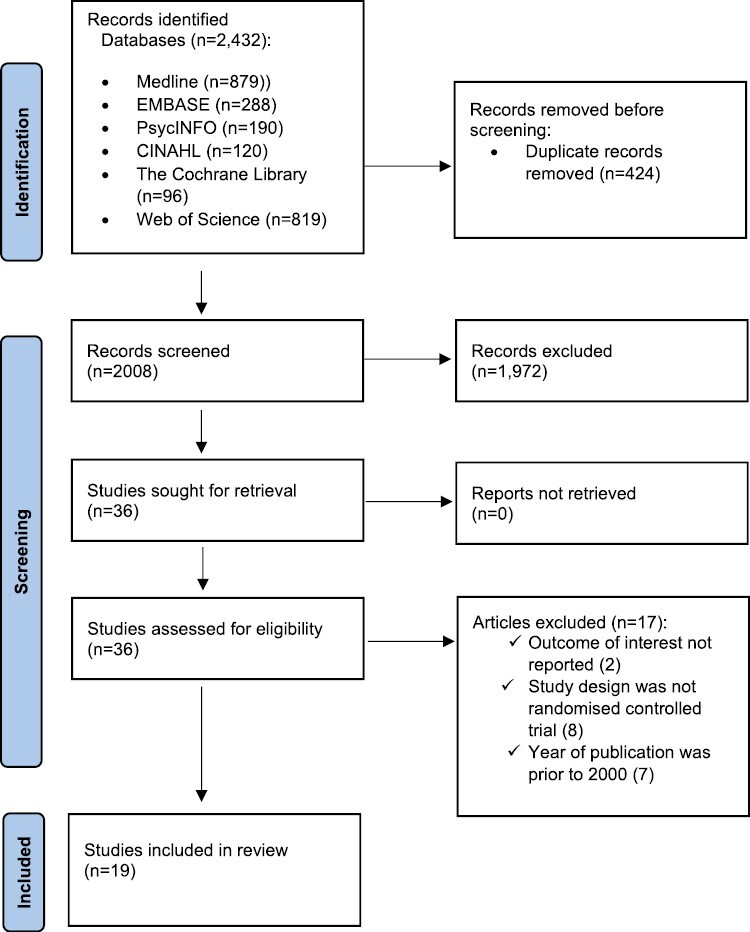
PRISMA flow diagram of studies included in the review.

An overview of the search results and the study coding process is outlined in Figure 1 using the PRISMA flow diagram. A total of 2432 citations were identified. After removing 424 duplicate citations, 2008 citations were screened for inclusion. After the full-text screening of 36 studies, 19 RCTs were included in the review.

### Study Characteristics

Ten studies were conducted in the United States.^12,13,19,28–34^ Three studies were conducted in India (*n* = 3).^35–37^ The remaining six studies were conducted in Denmark,^38^ Nigeria,^39^ Spain,^40^ China,^41^ Italy,^42^ and the Netherlands.^43^ Four studies were published between 2005 and 2009,^19,29,38,41^ five were published from 2010 to 2014,^13,32,35,36,40^ four studies were published between 2015 and 2019,^30,34,39,43^ and six studies were published from 2020 to 2021.^12,28,31,33,37,42^ Most studies used a two-arm RCT design (*n* = 16),^12,13,19,28–30,32–35,37–39,41–43^ and three studies used a three-arm RCT study design.^31,36,40^ Most of the included studies (*n* = 18) enrolled adults who smoked and showed interest in making a quit attempt. Nine studies had more limited inclusion criteria. One study each enrolled only Hispanic/Latin males,^28^ African Americans who were motivated to quit smoking,^30^ adolescents aged 12 to 17 years,^13^ individuals admitted for acute exacerbation of Chronic Obstructive Pulmonary Disease,^38^ and weight-concerned females.^19^ Two studies enrolled only males^28,37^ and one enrolled only females.^19^ Two studies enrolled ­people who smoked living with human immunodeficiency virus and were motivated to quit smoking.^32,33^ No studies focused on Indigenous populations. Characteristics are provided in Supplementary Material 2.

#### Methodological Quality

The methodological quality assessment scores for 11 studies were between 8 and 10^12,19,29–36,38–43^ out of a maximum score of 13 (range 7–11). Most studies did not or did not report blind treatments for the participants, therapist, and outcome assessors. The process of randomization and allocation concealments was not thoroughly described in the majority of the studies. Detailed assessments are available in Supplementary Material 3.

#### Components of the Comparators

Among the 16 studies that used a two-arm RCT study design^12,13,19,28–30,32–35,37–39,41–43^, nine studies compared the ­effectiveness of group-based interventions with usual ­smoking ­cessation care^12,13,28–30,33,38,39,41^. Other comparators included very brief smoking cessation advice (*n* = 1,^42^), self-help materials such as booklets and brochures (*n* = 2,^35,37^), and both brief smoking cessation advice and self-help materials (*n* = 1,^32^). In two studies, the same treatment was provided for the intervention and control groups, with the only difference being that treatment was tailored to the individual level in the control groups (*n* = 2,^19,34^). One study compared the effectiveness of group-based intervention with the addition of financial incentives for cessation in the treatment group.^43^

Among the studies that used a three-arm RCT study design (*n* = 3,^31,36,40^), two studies compared the effectiveness of the following three conditions: Standard care, individual tailored intervention, and group-based interventions.^36,40^ One study compared the effectiveness of the following three conditions: Brief tobacco intervention, a text message and color illustration of the advantages of remaining tobacco-free; a text and color illustration of the advantages of remaining tobacco-free only; or a standard smoking cessation intervention.^31^

#### Intervention Settings and Frequency

The interventions for nine trials were provided in a community setting,^13,28,31,35,36,39,41–43^ and nine trials were provided in a health care setting.^12,19,29,32–34,37,38,40^ The intervention for one study was provided in a university-based research clinic (*n* = 6). Face-to-face was the primary mode of intervention delivery in all studies. Group-based interventions were most often delivered by clinical psychologists (*n* = 6),^19,28,29,32,33,42^ followed by nurses (*n* = 3),^34,38,40^ physicians ( = 3),^34,35,40^ and substance abuse therapists (*n* = 3).^13,29,30^ The number of group-based sessions ranged from 12 sessions^34^ to 1 session.^28,31^ The mean number of group sessions used in trials were 5.31 ± 3.09. Sessions were delivered over a minimum period of six sessions over 2 weeks,^19^ to a maximum of eight sessions over 6 months.^33^ Four studies provided interventions weekly for the treatment period.^12,29,38,43^ Among trials with interventions delivered for two or more sessions, each session lasted from a minimum of 30 minutes^35,37^ to a maximum of 120 minutes per session^38,41^ [Supplementary Material 4].

#### Smoking Cessation Outcome Assessments

Fifteen studies used biochemical verification to confirm self-reported smoking abstinence (*n* = 15).^12,13,19,28–30,32–34,36,38,40–43^ Four studies reported only self-reported smoking abstinence.^31,35,37,39^ Smoking cessation at 6- to 12-month follow-up were evaluated in 14 studies (*n* = 14),^12,13,19,28–30,33,34,36,38,40–43^ while four studies reported smoking cessation rates for less than 6-month follow-up.^31,32,35,39^ Seven studies reported point prevalence abstinence (*n* = 7),^12,13,29,30,32,33,35^ and 12 studies reported continuous abstinence (*n* = 12).^19,28,31,34,36–43^

#### Theories and Treatment Guidelines Used to Inform Interventions

The Social Cognitive Theory ^44,45^ was used to inform intervention development in four studies (*n* = 4).^13,28,32,33^ Cognitive behavioral therapy^45^ was implemented in three studies (*n* = 3).^19,30,39^ Functional Analytic Psychotherapy and Acceptance and Commitment Therapy^46^ were followed to develop and implement group-based interventions in two studies.^12,29^ Group-based motivational interviewing ^46,47^ was used in three trials (*n* = 3).^31,40,42^ Included studies also referenced treatment guidelines and protocols of the Danish Cancer Society^46^; Quit Tobacco International group^47^; and the American Cancer Society^48^ as being used to inform the intervention protocol.^34,35,38^

### Aim 1

#### Most Frequently Occurring BCTs Used in Effective Group-Based Smoking Cessation Interventions

##### BCTs used.

A total of 28 BCTs from 13 BCT groups were identified (Table 1). Intervention protocols included a maximum of eight BCTs^13,28,30,41^ to a minimum of two BCTs.^34^ The average reported number of BCTs used in smoking cessation group-based interventions was 5.42 (SD = 2.0). The most frequently used BCTs in order of frequency were “information about health consequences” (*n* = 13),^13,30–33,35–41,43^ “problem solving” (*n* = 13),^13,19,28–30,32,33,35–37,40–42^ “avoidance/reducing exposure to cues for the behavior” (*n* = 12),^12,13,19,28,31–33,36,37,39–41^ “goal setting (outcome)” (*n* = 10),^12,13,28,30,32,33,36,38,40,42^ “reduce prompts/cues” (*n* = 7),^12,19,29,31–33,39^ and “pharmacological support” (*n* = 7).^12,28–30,33,34,38^

**Table 1.** Identified Behavior Change Technique and Sample Intervention Scenarios

BCT group	BCTs	Number of studies	Intervention examples	Reference
1. Goals and ­planning	1. Goal setting (outcome)	10	✓ Participants were supported and encouraged to set a quit date.	^12,13,28,30,32,33,36,38,40,42^
	2. Problem solving	13	✓ Participants were supported to identify and develop coping strategies for withdrawal symptoms, and cravings. ✓ Participants were taught how to control impulses and manage their negative emotions.	^13,19,28–30,32,33,35–37,40–42^
	3. Action planning	4	✓ Participants were supported and encouraged to make an achievable plan on how to quit smoking. ✓ Participants were supported and encouraged to prepare short- and long-term relapse prevention plans.	^12,28,40,41^
2. Feedback and monitoring	4. Feedback on behavior	2	✓ Participants were encouraged to discuss their smoking habits and feedback was provided.	^12,28^
	5. Self-monitoring of ­behavior	2	✓ Participants were learnt and encouraged on how to make records of circumstances for each cigarette smoked and monitor their quitting progress. ✓ Participants were empowered to monitor their treatment and progress.	^39,41^
	6. Monitoring of behavior by others without feedback	1	✓ Participants participated in an interpersonal exercise in which members shared feelings and experiences throughout treatment.	^29^
3. Social support	7. Social support (emotional)	2	✓ Family members and friends were encouraged to provide emotional support for participants.	^32,33^
	8. Social support (unspecified)	2	✓ Participants were guided and supported on how to stay quit through shared experiences amongst peers.	^30,43^
4. Shaping knowledge	9. Instruction on how to perform the behavior	1	✓ Participants were provided with detailed instructions on the proper use of NRTs.	^28^
5. Natural consequences	10. Information about health Consequences	13	✓ A range of information was provided regarding the health risks of smoking, the tobacco-related burden of disease, and the benefit of smoking cessation.	^13,30–33,35–41,43^
	11. Information about social and environmental consequences	5	✓ The risk of passive smoking and health hazard to other family members were discussed.	^13,35,37,38,41^
	12. Information about emotional consequences	1	✓ Participants were provided with an education on the psychological impact of smoking.	^29^
6. Comparison of behavior	13. Demonstration of the behavior	2	✓ Role-playing was used to demonstrate how to avoid or deal with social influence such as when a family member or friend offers the adolescent tobacco.	^13,41^
	14. Comparative imagining of future outcomes	1	✓ Participants were encouraged to share their quitting experiences, and the challenges they face with the group members.	^41^
7. Associations	15. Reduce prompts/cues	7	✓ Practical sessions and information were provided on how to avoid triggers to use tobacco.	^12,19,29,31–33,39^
8. Repetition and substitution	16. Behavioral practice/rehearsal	3	✓ Participants rehearsed metaphors and experiential exercises on how to avoid and respond to situations where there is increased pressure to smoke.	^12,13,42^
	17. Behavior substitution	1	✓ Participants were encouraged to discuss and focus on religion/spirituality, and family/collectivism.	^30^
9. Comparison of outcomes	18. Credible source	1	✓ Participants attended a session where successful quitters shared their experiences. ✓ Credible individuals such as dentists provide education on the risk of smoking.	^13^
.	19. Comparative imagining of future outcomes	1	✓ Participants were provided with a colored illustration to demonstrate a life without tobacco.	^31^
10. Reward and threat	20. Material incentive (behavior)	1	✓ Participants were incentivized for each attended group session.	^34^
	21. Reward (outcome)	3	✓ Abstinent participants were provided with gift vouchers for their achievement.	^34,36,43^
	22. Social reward	1	✓ Participants who attended group sessions and remained abstinent were recognized to encourage them and other group members.	^41^
	23. Self-reward	1	✓ Participants were encouraged to reward themselves for positive outcomes.	^39^
11. Regulation	24. Pharmacological support	7	✓ Participants were offered pharmacological treatment with smoking cessation medications such as NRT or bupropion.	^12,28–30,33,34,38^
12. Antecedents	25. Avoidance/reducing exposure to cues for the behavior	12	✓ Strategies on how to reduce environmental cues were discussed.	^12,13,19,28,31–33,36,37,39–41^
	26. Restructuring the social environment	2	✓ Social cues for smoking and strategies to reduce them were discussed such as handling cigarettes.	^29,30^
13. Self-belief	27. Verbal persuasion about capability	2	✓ Participants were motivated by affirming their autonomy to improve self-efficacy.	^28,42^
	28. Self-talk	3	✓ Participants were encouraged to self-construct their thoughts on the benefit of smoking and controlling impulses.	^29,30,39^

BCTs = Identified Behavior Change Technique

Some examples of how the most frequently used BCTs were operationalized in interventions are:

- Information about health consequences: The smoking cessation counselors discussed topics, such as tobacco-related facts, raise awareness through photographs of diseased body parts using a flip chart, illness narratives, myths and facts, and benefits of quitting tobacco. Session coordinators then elicit group discussions about the negative aspects of tobacco use and the health benefit of quitting.^31,35^
- Problem solving: The sessions included a discussion of group members’ experience, withdrawal symptoms and measures taken to overcome them, and coping strategies/prevention of relapse.^35^ A series of metaphors and experiential exercises led by the counselor was practiced as a group with discussion and feedback afterwards on resolving unwanted thoughts, emotions, and sensations that could detour their journey.^12^
- Avoidance/reducing exposure to cues for the behavior: Counselors role-played situations involving social influence, such as when a family member or friend offers tobacco. Each participant had the opportunity to observe behaviors modeled by others and to enact behavior.^13^
- Goal setting (outcome): Participant encouraged to share their progress toward their goals with the group. Group members reviewed their action plans and were encouraged to cut back on their smoking, set a quit date, use NRT, and take other committed actions toward quitting and remaining abstinent.^12^

##### Intercoder agreement:

Substantial intercoder agreement (kappa score of 0.61–0.80) was observed for BCT coding in three studies.^19,35,38^ The intercoder agreement rate for BCT identification of the remaining 16 studies was ≥ 0.81 indicating an almost perfect agreement.^12,13,28–34,36,37,39–43^ The mean kappa score across all studies was found to be 0.89 ± 0.09, indicating a good over all agreement rate between coders (Supplementary Material 4).

#### Most Frequently Occurring BCTs Used in Effective Group-Based Smoking Cessation Interventions

**Figure 2.**
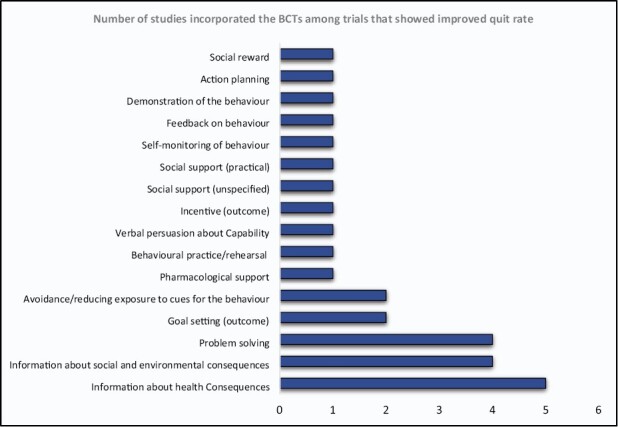
The frequency of behavioral change techniques appeared in studies that showed improved quit rate.

Eleven studies did not report statistically significant differences between the comparator and treatment groups at the last assessment follow-up (*n* = 11).^12,13,19,28–34,40^ Eight trials reported statistically significant differences between the intervention and comparator groups at the final assessment follow-up (*n* = 8).^35–39,41–43^ Among these eight studies, group-based interventions were reported to increase rates of smoking cessation from a minimum of two-fold, (AOR = 1.93, 95% CI = 1.31 to 2.85)^43^ to a maximum of more than six-fold (AOR = 6.42, 95% CI = 2.46 to 13.28)^41^ [Supplementary Material 2]. The most frequently used BCTs among the eight studies (*n* = 8)^35–39,41–43^ that identified a statistically significant smoking cessation rate between the treatment and comparator arm were “information about health consequence” (*n* = 5)^35,37,38,41,43^ followed by “information about social and environmental consequence” (*n* = 4)^35,37,38,41^ and “problem solving” (*n* = 4).^35,37,41,42^Figure 2 presents the most frequently occurring BCTs among effective interventions [Supplementary Material 4].

Among the eight studies that demonstrated the effectiveness of group-based smoking cessation interventions (*n* = 8),^35–39,41–43^ six were implemented in community settings, including workplaces (*n* = 6),^35,36,39,41–43^ and two were implemented in health care settings (*n* = 2).^37,38^ Interventions were implemented between two^37^ and seven^43^ sessions. Half of the eight trials provided five sessions once or twice per week.^36,38,39,41^ Intervention sessions lasted 120 minutes in three of the eight studies (*n* = 3).^38,41,42^ Six of the eight studies measured smoking cessation between 6 and 12 months (*n* = 6).^36–38,41–43^ Interventions were delivered by nurses,^38^ clinical psychologists,^42^ physicians,^35^ dentists,^36^ researchers,^37,39^ professional coaches with experience in smoking cessation group training,^43^ and health education professionals.^41^

### Aim 2

#### The Effectiveness of Group-Based Smoking Cessation Interventions on Smoking Cessation Rates at 6-Month Follow-Up

**Figure 3.**
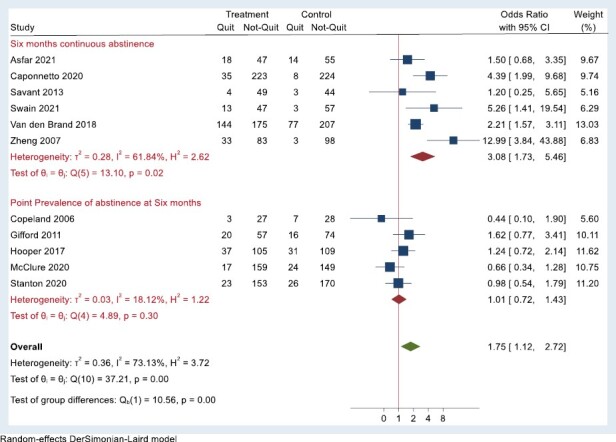
Effectiveness of group-based interventions split for outcome measure at 6-month follow-up.

Eleven of the nineteen included studies reported smoking cessation outcomes at 6-month follow-up. The pooled rate of smoking cessation among these studies was higher among participants randomized to a group-based intervention arm compared to any comparator arm (OR = 1.75, 95% CI = 1.12 to 2.72, *p* < .001). Moderate heterogeneity was seen in the overall pooled result (*I*² = 73.1%), and we, therefore, conducted a sensitivity analysis by grouping outcomes based on type (continuous abstinence vs. point prevalence of abstinence) to try to account for some of this heterogeneity. The pooled rate of continuous abstinence at 6 month was found to be higher in the treatment arm as compared to comparator arm (OR = 3.08, 95% CI = 1.73 to 5.46, *p* = .02, *I*² = 61.8%). When point prevalence abstinence was used as the outcome measure, we did not identify a significant treatment effect (Figure 3).

**Figure 4.**
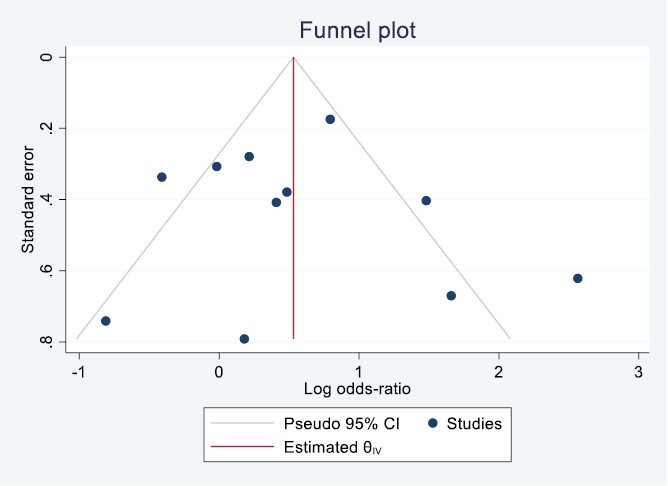
Publication bias of included studies.

The funnel plot for the 11 studies that reported smoking cessation rates at 6 months illustrated symmetrical ­distribution. Regression-based Egger’s test for small study effect was not significant (Figure 4).

### Aim 3

#### Individual BCTs and BCT Domains Related to Effective Group-Based Smoking Cessation Interventions at 6-Month Follow-Up

Meta-analysis using the random-effect model was performed to estimate the effectiveness of BCT domains and specific BCTs that were used in at least two trials that reported smoking cessation outcomes at 6-month follow-up. A total of 25 meta-analyses were conducted—10 meta-analyses for BCT domains and 15 meta-analyses for specific BCTs. The summaries are presented in Table 2. The forest plots and funnel plots are at Supplementary Materials 5 and 6.

**Table 2.** Meta-Analyses: Associations Between Individual Behavior Change Techniques and 6-Month Smoking Cessation

BCTs	Number of studies	Odds ratio (95% CI) *p* value	*p* value	I² (%)	Egger test *p* value
Goals and planning	**9**	**1.88(1.10 to 3.22)**	.02*	**74.2**	.039*
Goal setting (outcome)	6	1.34(0.80 to 2.23)	0.27	64.4	0.715
Problem solving	8	2.10(1.10 to 4.02)	.02*	72.5	0.838
Action planning	3	2.16(0.49 to 9.54)	0.31	88.7	.001*
Feedback and monitoring	**4**	**1.94(0.71** to**5.29)**	**0.20**	**83.2**	.001*
Feedback on behavior	2	0.97(0.43 to 2.15)	0.94	58.1	0.409
Self-monitoring of behavior^¹^	1	—	—	—	—
Monitoring of behavior by others without feedback^¹^	1	—	—	—	—
Social support	**3**	**1.46(0.87** to **2.45)**	**0.16**	**70.2**	.009*
Social support (emotional)^¹^	1	—	—	—	—
Social support (unspecified)	2	1.72(0.98 to 3.03)	.06	67.7	0.573
Shaping knowledge^¹^	1	—	—	—	—
Instruction on how to perform the behavior^¹^	1	—	—	—	—
Natural consequences	**8**	**1.75(1.05** to **2.91)**	.03*	**75.1**	**0.171**
Information about health Consequences	5	2.58(1.20 to 5.55)	.02*	76.7	0.489
Information about social and environmental consequences	2	8.55(3.50 to 20.88)	.001*	0.0	0.457
Information about emotional consequences^¹^	1	—	—	—	—
Comparison of behavior^¹^	**1**	—	—	—	—
Demonstration of the behavior^¹^	1	—	—	—	—
Comparative imagining of future outcomes^¹^	1	—	—	—	—
Associations	**3**	**1.07(0.62** to **1.85)**	**0.80**	**25.4**	**0.335**
Reduce prompts/cues	3	1.07(0.62 to 1.85)	0.80	25.4	0.335
Repetition and substitution	**3**	**1.50(0.56** to **3.97)**	**0.42**	**84.7**	**0.359**
Behavioral practice/rehearsal	2	1.69(0.26 to 10.75)	0.58	92.2	0.323
Behavior substitution^¹^	1	—	—	—	—
Reward and threat	**5**	**2.13(1.19** to **3.82)**	.01*	**69.3**	**0.557**
Reward (outcome)	2	2.15(1.54 to 3.00)	.001*	0.0	0.663
Social reward^¹^	1	—	—	—	—
Regulation	**5**	**1.11(0.82** to **1.50)**	**0.49**	**4.8**	**0.520**
Pharmacological support	5	1.11(0.82 to 1.50)	0.49	4.8	0.520
Antecedents	**9**	**1.50(0.90** to **2.52)**	**0.12**	**68.6**	**0.256**
Avoidance/reducing exposure to cues for the behavior	7	1.59(0.75 to 3.37)	0.22	76.1	0.395
Restructuring the social environment	2	1.36(0.88 to 2.12)	0.17	0.0	0.566
Self-belief	**4**	**1.84(1.07** to **3.16)**	.03*	**56.6**	**0.300**
Verbal persuasion about capability	2	2.58(0.90 to 7.36)	.08	71.3	0.375
Self-talk	2	1.36(0.88 to 2.12)	0.17	0.0	0.566

Bold text indicates meta-analysis results for domains. BCTs = Identified Behavior Change Technique

^¹^Insufficient number of studies to perform meta-analyses (less than two studies).

From 13 BCT domains identified in the included studies, 10 domains appeared in at least two trials that reported smoking cessation outcomes at 6-month follow-up—“Goals and planning,” “Feedback and monitoring,” “Social support,” “Natural consequences,” “Associations,” “Repetition and substitution,” “Reward and threat,” “Regulation,” “Antecedents,” and “Self-belief.” The inclusion of the ­following four BCT domains “Goals and planning,” “Natural consequence,” “Reward and threat,” and “Self-belief” were significantly associated with increased intervention effectiveness at 6 months compared with comparison arms (Table 2).

From a total of 28 specific BCTs identified in the included studies, 15 BCTs appeared in at least two trials that reported smoking cessation outcomes at 6-month follow-up. These BCTs are the following: “Goal setting (outcome),” “Problem solving,” “Action planning,” “Feedback on behavior,” “Social support (unspecified),” “Information about health consequences,” “Information about social and ­environmental consequences,” “Reduce prompts/cues,” “Behavioral practice/rehearsal,” “Reward (outcome),” “Pharmacological support,” “Avoidance/reducing exposure to cues for the behavior,” “Restructuring the social environment,” “Verbal persuasion about capability,” and “Self-talk.” Inclusion of the following four BCTs “Problem solving,” “Information about health consequences,” “Information about social and environmental consequences,” and “Reward (outcome)” were significantly associated with increased intervention effectiveness at 6 months compared with comparison arms. (Table 2) The inclusion of “Information about social and environmental consequences” demonstrated the highest effect on smoking cessation during 6-month follow-up (OR = 8.55, 95% CI = 3.50 to 20.88, *p* < .001). The funnel plot illustrated a symmetrical distribution and the regression-based Egger test for small-study effect was not significant (Table 2).

## Discussion

Consistent with the WHO Framework Convention on Tobacco Control,^4^ group-based smoking cessation programs offer an efficient way of reaching and supporting a people who smoke to quit. This included the need for comprehensive tobacco control to support smoke free behaviors. This review identified components of effective group-based smoking cessation interventions. It is the first to apply the BCT taxonomy to map interventions used in group-based smoking cessation studies to provide critical information for researchers and those delivering health programs about the components of effective group-based interventions that can be replicated in future research and healthcare delivery.

Group-based smoking cessation programs were associated with higher odds of quitting compared to participants receiving a comparator intervention such as usual care or self-help martials. This is consistent with a 2017 Cochrane review that concluded that group-based therapy is more effective for helping people quit smoking than self‐help, and other interventions, such as behavioral support or care as usual.^14^ Sub-group analysis illustrated three-fold higher odds of smoking cessation in the group-based intervention arm using 6 months continuous abstinence as the outcome. We did not identify significant differences for group-based programs and comparators when using point prevalence abstinence at 6-month follow-up. Analyses may have been limited by study power to detect significant differences, but this does not provide evidence that group-based cessation supports were, or were not, effective when smoking cessation was measured using point prevalence at 6 months. The finding of significant improvements in smoking cessation among group-based cessation supports were reported previously but have not been reported based on the type of outcome measures.^14^ Similar to the 2017 Cochrane review, we were unable to determine the relative effectiveness of group-based interventions compared to intensive individual counseling, as there were insufficient studies for meta-analysis. This is an important consideration given the potential cost-effectiveness of group-based versus individual counseling.^14^

The review identified an average of 5.42 BCTs in group-based interventions in contrast, a systematic review of internet-based smoking cessation interventions found an average of 6.6 BCTs per intervention arm across 45 included studies, while a systematic review of digital interventions for pregnant women found an average of 10 BCTs per intervention across 12 included studies.^17^ Given meta-regressions have suggested that interventions using larger numbers of BCTs produced the greatest effects, increasing the number of BCTs in group-based interventions may contribute to increased intervention effectiveness.^49^ The most frequently occurring BCTs used across all interventions were the provision of information about health consequences, problem solving (which relates to developing techniques to avoid smoking during times when the desire to smoke is strong), avoidance/reducing exposure to cues for the behavior, and goal setting (setting a quit date). Less than half of the interventions reported using pharmacological support, which is notable given that combining behavioral interventions such as counseling and pharmacotherapy is considered best practice for smoking cessation.^49,50^ It is also notable that few interventions reported the use of social support to aid quitting given the format of group programs offers participants a unique opportunity for social learning such as sharing skills and knowledge and providing mutual support.

Group-based smoking cessation interventions are being increasingly considered.^11,51^ Of the eight included studies that demonstrated effectiveness of a group-based smoking cessation program, the majority were administered in community settings over five sessions provided once or twice per week. BCTs found to be significantly associated with increased intervention effectiveness at 6-month follow-up included the provision of information about health consequences, the provision of information about social and environmental consequences, Reward (outcome), and problem solving. It is possible that using this suite of complementary and effective BCTs could enhance the effect of group-based smoking cessation programs.

Our findings vary with findings reported in systematic reviews examining use of BCTs in smoking cessation interventions; however, group-based, and one-to-one interventions were often not separated on these reviews. For example, Fujii et al.^52^ found social support (emotional), instruction on how to perform behavior, and goal setting (behavior) to have positive effect on smoking cessation (along with adherence to medication and correct inhaler use) among patients with chronic respiratory diseases. Similarly, Black et al.^53^ while reporting evidence on effects of BCT clusters and individual BCT techniques on smoking behavior found that high number of BCT components to be more effective in helping participants quit smoking in the general population, with prompting commitment, social reward, identity associated with changed behavior to be most effective when the mode of delivery was interpersonal either via individual or group-based intervention.^53^ Bartlett and colleagues^54^ identified that facilitating action planning or developing treatment plan, prompting self-recording, advising on methods of weight control, and advise on/facilitating use of social support were effective BCT techniques among individuals with COPD.^54^ However, the above-mentioned reviews present evidence from both individual-based and group-based delivery of BCT interventions.

The meta-analysis illustrated an approximate two-fold improvement in the odds of smoking cessation for studies that incorporated the BCT domains “Goals and planning,” “Natural consequence,” “Reward and threat,” and “Self-belief” as compared to the control groups. Incorporating “Information about social and environmental consequences” improved rate of smoking cessation by more than eight-fold. In addition, “Problem solving,” “Information about health Consequences,” and “Reward (outcome)” were associated with a 2 to 3 times higher rates of smoking cessation. The use of BCTs from domains “Goals and planning” and “Natural consequences,” were found to improve smoking cessation rates when included in Internet-based interventions.^17^ In addition, “Problem solving,” “Information about health Consequences,” and “Information about social and environmental consequences” are commonly part of evidence-based individual smoking cessation therapy.^6,55^ Therefore, incorporating these BCTs in individual or group-based smoking cessation programs delivered either face-to-face or in Online platforms holds significant potential in improving the success of quit attempts.

By using the BCT taxonomy, the findings from this study can be replicated for group-based smoking cessation programs as well as other health research targeting behavior changes. The CONSORT (Consolidated Standards of Reporting Trials) statement on reporting randomized trials recommends the reporting of the details of interventions involved in the trial to allow replication of the study.^20^ Similarly, various guidelines such as The Intervention Description and Replication (TIDieR) guideline,^56^ the UK Medical Research Council’s (MRC) Framework for the Development and Evaluation of Complex Interventions,^22^ and Guidance for reporting intervention development studies in health research (GUIDED)^57^ recommend including details of interventions in reporting randomized trials so as to allow a clear replication. While group-based smoking cessation interventions appear to hold significant potential, there is limited evidence on effectiveness of BCT technique-oriented interventions delivered in group settings. Completeness of description of BCT components included in published interventions are critical for comprehending the effectiveness of those components better. Lorencatto and colleagues^58^ highlighted that published descriptions of behavioral smoking cessation interventions may not include more than half of the BCT components specified in the intervention manual.

Our study identified no published RCT that used BCT components were conducted with Indigenous peoples. Therefore, there is a critical need for evidence on Indigenous peoples specific BCT-oriented smoking cessation intervention. Connection to community and culture is critical to Indigenous health among many Indigenous peoples worldwide, with community often being a source of social, emotional, and spiritual support.^59^ Therefore, BCT-oriented group-based smoking cessation interventions in the community settings may be effective in advancing Indigenous health. Such interventions ­warrant rigorous evaluation as well.

### Strengths and Limitations

A significant strength of the review is the systematic review approach that implemented PRISMA and systematic review protocol, including the use of independent coders at each step of the data extraction process. The meta-analysis ­included the results of 19 studies including and updates previous estimates of the effectiveness of group-based smoking cessation programs provided in 2017 Cochrane review. Several limitations should be noted. First, coding of BCTs relied on published literature. Given the recency of development of the 93 BCT taxonomy, it may be that studies failed to describe BCTs in a way that facilitated their coding, or intervention descriptions could have left out some components of interventions that were assumed not critical by authors. This would reflect differences in reporting of BCTs, rather than use of BCTs. Coding of intervention materials in their entirety (eg, intervention deliver manuals, review of patient materials) would give more accurate indication of the types of BCTs used. This highlights the importance of intervention developers and researchers to report on the use of active ingredients in their smoking cessation interventions to increase replicability.

Second, the small overall number of studies means that interventions with different comparator arms were combined in meta-analysis. There are limitations to this approach, with meta-analyses and meta-regressions that do this ignoring potential confounders. For example, some comparator arms were more intensive than others and, therefore, any BCT shared between intervention and comparator groups may have cancelled each other out. It remains a priority that developing interventions which can truly isolate the effectiveness of individual techniques is still a priority for the future of BCT research. Finally, studies included in the review are heterogeneous with varied populations and implementation settings. Further work is needed to better understand the BCTs that work for different population groups, and how BCTs should be implemented to be most effective.

## Conclusions

Group-based smoking cessation interventions double the odds of smoking cessation at 6-month follow-up compared to usual care individual-based treatments. BCTs that increase likelihood of effectiveness of group-based programs include provision of information about health consequences, the provision of information about social and environmental consequences, and the use of problem solving.

## Supplementary Material

A Contributorship Form detailing each author’s specific involvement with this content, as well as any supplementary data, are available online at https://academic.oup.com/ntr.

ntad068_suppl_Supplementary_Material_S1

ntad068_suppl_Supplementary_Material_S2

ntad068_suppl_Supplementary_Material_S3

ntad068_suppl_Supplementary_Material_S4

ntad068_suppl_Supplementary_Material_S5

ntad068_suppl_Supplementary_Material_S6

## Data Availability

All relevant materials and data supporting the findings of this review are included within the manuscript.
