# Supplementary material for: What Are the Effective Components of Group-Based Treatment Programs For Smoking Cessation? A Systematic Review and Meta-Analysis
Source: Nicotine Tob Res. 2023 Apr 27;25(9):1525–37. doi: 10.1093/ntr/ntad068 (PMC10439487; doi:10.1093/ntr/ntad068)
Supplement: ntad068_suppl_Supplementary_Material_S1 [file ntad068_suppl_supplementary_material_s1.docx]

**Search terms**

**#1** ‘Tobacco Use Cessation’ OR ‘Smoking Cessation’ OR ‘Smoking’ OR ‘smoking cessation’ OR ‘Smoking cessation’ OR ‘tobacco products’ OR ‘Tobacco Use’ OR Smoking OR nicotine OR tobacco OR cigar OR hookah OR quitting OR cessation OR stopping OR Cigarette OR Cigar

**AND**

**#2** group OR ‘group therapy’ OR ‘group therapies’ OR ‘Group therapy' OR ‘Group Psychotherapy’ OR ‘Support Group’ OR ‘Self Help Groups’ OR Groups OR Support OR ‘Support Groups’ OR ‘Self-Help Group’ OR 'Cognitive therapy' OR Psychotherapy [OR](https://www.cochranelibrary.com/advanced-search/mesh#0) 'Behaviour therapy' OR 'Behavior therapy' OR ‘Behavior Change Technique’ OR ‘Behavior Modifications’ OR Therapy OR Behavior OR ‘Behavior Therapies’ OR ‘Behavior Treatment’ OR Behavior OR ‘Behavior Modification’ OR ‘Behavior Change Techniques’ OR ‘Behavior Change’

MEDLINE search

| 1. | smoking Cessation.mp. or Smoking/ or Smoking Cessation/ or "Tobacco Use Disorder"/ |
| --- | --- |
| 2. | Tobacco Cessation.mp. or "Tobacco Use Cessation"/ |
| 3. | hookah.mp. or Smoking Water Pipes/ |
| 4. | Cigar.mp. |
| 5. | pipe tobacco.mp. |
| 6. | chewing tobacco.mp. |
| 7. | Tobacco/ |
| 8. | tobacco products/ or tobacco, smokeless/ or tobacco, waterpipe/ |
| 9. | group-based.mp. |
| 10. | group therapies.mp. or Psychotherapy, Group/ |
| 11. | group therapy.mp. |
| 12. | Group Psychotherapy.mp. |
| 13. | Self-Help Group.mp. or Self-Help Groups/ |
| 14. | Support Group.mp. |
| 15. | 1 or 2 or 3 or 4 or 5 or 6 or 7 or 8 |
| 16. | 9 or 10 or 11 or 12 or 13 or 14 |
| 17. | 15 and 16 |
| 18. | limit 17 to (english language and humans and randomized controlled trial) |
