# Supplementary material for: What Are the Effective Components of Group-Based Treatment Programs For Smoking Cessation? A Systematic Review and Meta-Analysis
Source: Nicotine Tob Res. 2023 Apr 27;25(9):1525–37. doi: 10.1093/ntr/ntad068 (PMC10439487; doi:10.1093/ntr/ntad068)
Supplement: ntad068_suppl_Supplementary_Material_S2 [file ntad068_suppl_supplementary_material_s2.docx]

**Supplementary table: Overview of included studies.**

| First author and year | Country | **Study design** | Sample size: | Inclusion/ Exclusion criteria | Outcome definition | Components | Comparator | Main findings  6 months | Main findings  >or<6 months |
| --- | --- | --- | --- | --- | --- | --- | --- | --- | --- |
| Asfar 2021 | USA | RCT | Start of trial  Total: 134  Intervention group: 65  Control group: 69  Analysed  Total:  Intervention group: 65  Control group: 69 | Inclusion: ≥18 years old, Hispanic/Latin, male, smoking ≥5 cigarettes/day for the past year, interested in making a quit attempt in the next 30 days, access to a telephone, and not planning to move in the next 6 months.  Exclusion: contraindication to NRT and the inability to understand consent procedures. | Self-reported prolonged abstinence (no smoking, not even a puff) verified by ECO <10 parts per million at the 6-month follow-up. | The content of the group counseling session included preparing to quit, setting quit attempt, surviving the first days without smoking, developing long-term relapse prevention, and using NRT properly. | Standard care | No significant difference between groups in verified prolonged abstinence rates in the treatment group and the control (27.7% and 20.3% respectively at 6 months (p-value =0.315)). | No significant difference between groups in the verified prolonged abstinence rates in the treatment and control (44% and 38% respectively at 3 months (p-value =0.571)) |
| Borglykke 2008 | Denmark | RCT | Start of trial Total: 223, Intervention group: 121, Control group: 102  Analysed Total: 223, Intervention group: 121, Control group: 102 | Individuals admitted for acute exacerbation of Chronic obstructive pulmonary disease (COPD) | Self-reported abstinence verified with carbo-hemoglobin measurement. | Intervention contained  information on smoking cessation and supporting to set up a quit date. | Standard care | Not measured | After 1 year, 36 (30%) patients in the intervention group were abstinent compared with 13 (13%) patients in the control group [OR= 2.83 (1.40–5.74)]. |
| Caponnetto 2020 | Italy | RCT | Start of trial: Total: 656, Intervention group: 328, Control group: 328  Analysed Total: 490, Intervention group: 258, Control group: 232 | Eligible participants adults employed in Italian supermarkets, regular smokers, had smoked cigarettes during the preceding month, had a concentration of ECO >10 ppm, aged between 18 and 65 years. | Abstain from cigarette smoking during this period with ECO measurements ≤10 parts per million. | Group motivational interviewing counseling was used to elicit motivation to change by helping clients explore and resolve ambivalence. Emphasis was placed on creating a collaborative relationship and affirming the client’s autonomy regarding change.  Therapists evoked motivation for change by drawing on client’s goals and values. | four sessions of very brief advice | The Continuous Quit Rate was higher for the smoking cessation counseling group than for the very brief advice group during weeks 9 to 24 (13.4% vs. 3.4%) with p<0.001. | The Continuous Quit Rate was higher for the smoking cessation counseling group than for the very brief advice group during weeks 9 to 12 (17.5% vs. 3.6%) and weeks 9 to 52 (10.3% vs. 3.1%) with p<0.001.  Overall OR=2.34 (1.87-2.64) |
| Copeland 2006 | USA | RCT | Start of trial: Total: 76, Intervention group: 36, Control group: 40  Analysed: Total: 65, Intervention group: 30, Control group: 35 | Participants included if they are weight-concerned (i.e., endorsed use of smoking for weight control and fear of post cessation weight gain) female smokers, who smoked at least 10 cigarettes per day for at least 1 year, had a body mass index (BMI) greater than 18, and ECO >10 ppm.  Participants with active substance use disorders, major affective disorders, eating disorders, or psychotic disorders were excluded. | Breath CO monitor and used a cut-off level of <10 ppm to confirm non-smoking status. | Educational information on health consequences, nicotine addiction, and withdrawal, environmental management of smoking cues, relapse prevention, and cognitive restructuring beliefs about smoking.  The clinical psychologists discussed with group participants situations that were high-risk for smoking relapse, including weight concerns and negative mood, and how to successfully cope with them. | The same treatment tailored at individual level. | Point prevalence abstinence rates were higher among individual tailored (21.1%) than group participants (8.3%) (p < 0.05). | Continuous 6- abstinence  show a nonsignificant trend for higher abstinence rates among individual tailored participants (17.5%) than among group participants (8.3%) (p < 0.10). |
| **Gifford 2011** | USA | RCT | Start of trial: Total: 303,Intervention group: 130, Control group: 173  Analysed: Total: 167, Intervention group:77, Control group: 90 | Inclusion: Smoke ≥15 cigarettes per day for 12 months with FTND score of ≥5.  Exclusion: Diagnosis for alcohol or drug dependence; anorexia nervosa; current on NRT, and fluoxetine.  Diagnosis of major depressive disorder, bipolar disorder, anxiety disorder, age <18 years, seizure disorder, pregnancy. | Seven-day point prevalence confirmed with ECO. | Elicit and reinforce positive behavioural steps taken by the client.  Discussing negative feelings- would be linked to the discomfort of refraining from smoking.  A discussion of external triggers.  Engage in exposure to the thoughts and feelings that might ordinarily lead to smoking, while learning to respond to those cues.  Mindful breaks without smoking, and ultimately to handling cigarettes, lighters, and other smoking-related items.  Interpersonal exercises in which members shared feelings and experiences throughout treatment. | Standard care | The 6-month assessment was not significantly different, with 26.2% quit in the treatment group versus 18.2% quit in the medication alone group, p=.162. | Using data posttreatment (10 weeks post quit date), 7-day point prevalence quit status differed significantly, with 50.0% quit in the treatment group compared with 27.9% quit in the bupropion alone group. |
| Hooper 2017 | USA | RCT | Start of trial: Total: 342, Intervention group: 168, Control group: 174  Analysed: Total: 282, Intervention group: 142,Control group: 140 | Include: (1) self-identification as African American; (2) currently smoked five or more cigarettes per day or an expired carbon monoxide level of ≥8 ppm; (3) ages 18–65 years; (4) able to read fifth- to sixth-grade English; (5) permanent contact information; (6) ability to attend group sessions; and (7) motivated to quit smoking (6 on a 1–10 scale).  Exclude: receiving current smoking cessation, alcohol, or illicit drug treatment, or contraindications for nicotine patch use (acute cardiac condition, pregnant, or breastfeeding). | Smoking abstinence was verified biochemically using saliva cotinine, using 7 ng/mL as the cut point. Breath carbon monoxide verified abstinence at the end of treatment (due to nicotine patch use) with <8 ppm as the cut point. | Discussed smoking and health, self-motivation, and goal setting. In the culturally specific CBT condition, the history of medical and research distrust and potential concerns about pharmacotherapy was also discussed. | Standard care | No significant differences in the odds of 7-day abstinence at the 6- month follow-up. OR=1.26, 0.76-2.08.  Intent-to-treat analysis  7-day abstinence at the 6- month was 26.2% and 22% for the treatment and control groups respectively. | Significantly greater odds of 7-day abstinence in the culturally specific condition at the end-of-therapy (P = .05) (OR 1.56, 1-2.42) and 3-month follow-up (OR 1.92, 1.19-3.10) in intent-to-treat analyses. |
| Kumar 2012 | India | RCT | Start of trial: Total: 400, Intervention group: 200, Control group: 200  Analysed: Total: 366, Intervention group: 181, Control group: 185 | Inclusion: men, aged 20–40 years, resident of the village, current user of any form of  tobacco, and willing to participate in the study and provide informed consent.  Exclusion: Men who were planning to migrate in the next 3 months and older than 40 years. | self-reported point prevalence abstinence (no tobacco use in the past 7 days) at 2 months after recruitment. | The contents of the sessions included tobacco-related health problems, benefits of quitting, and coping strategies for withdrawal symptoms.  Components of first session covered topics like tobacco related facts, raising consciousness through photographs of diseased body parts using a flip chart, illness narratives, myths and facts, benefits of quitting tobacco, and plan for quitting tobacco.  The second session included discussion of their experience, withdrawal symptoms and measures to overcome them, coping strategies/prevention of relapse, and hazards of second-hand smoke. | Self-help material | Not measured | Significant difference in the self-reported point prevalence abstinence from tobacco use at 2 months in the intervention and control groups (13.8% and 6.5%, respectively). Or= 2.66 (1.19–5.9), p-value 0.016. |
| Little 2020 | USA | **RCT-** three-group clustered randomized clinical trial | Analysed: Total: (1) Brief Tobacco Intervention (BTI) + Airman’s Guide to Remaining Tobacco Free (AG), - 1283, (2) AG intervention, - 688, (3) standard smoking cessation intervention, - 640 | 18 years of age and understanding the consent process in English. | tobacco use was defined as any tobacco use at the 3-month follow-up. | Brief Tobacco Intervention:  Eliciting participation through the principles of motivational interviewing.  Enhanced discussions about the negative aspects of tobacco use and placed a greater emphasis on Airmen’s control.  Discussing positive social activities on base could help Airmen avoid situations where there might be pressured to use tobacco.  The Airmen’s Guide to Remaining Tobacco Free:  he text covers the advantages of remaining tobacco free after BMT, and the opportunity the ban provides to begin a life without tobacco, focusing on cigarettes and smokeless tobacco. The text is supplemented by images created specifically for Airmen and designed to reinforce the messages that smoking (1) is responsible for more fatalities than combat, (2) conveys a negative image to civilians, and (3) impedes military readiness and promotion through the ranks. | The control group received standard care | Not measured | No significant difference by condition in the use of tobacco products at 3 follow-up (*p* = .454) |
| McClure 2020 | USA | RCT | Start of trial: Total: 450, Intervention: 224, Control: 226  Analysed: Total: 349, Intervention: 176, Control: 173 | Smoker in the past year, at least 18 years old, smoked at least 10 cigarettes a day, wanted to quit smoking in the next month, were not currently using treatment to quit smoking, had no contraindications for NRT, and had no cognitive or physical impairment precluding participation. | self-reported 30-day point prevalence abstinence (PPA) at 12 months. Secondary outcomes included biochemically confirmed PPA at 1-year and 30-day PPA (self-reported and biochemically confirmed) at 6 months. | Each acceptance and commitment  therapy (ACT) session focused on two broad themes: acceptance (acceptance, awareness, and being present) and commitment (values, committed action). Participants learned these concepts through a series of metaphors and experiential exercises led by the counsellor.  Learn how to not be distracted (i.e., unwanted thoughts, emotions, and sensations) that could detour their journey.  Exercises were practiced as a group with discussion and feedback afterwards.  Each session ended with an action plan review during which participants were encouraged to cut back on their smoking, set a quit date, use NRT, and take other committed actions toward quitting and remaining abstinent. | Standard care | At 6-month follow-up, self-reported 30-day PPA rates were lower in the ACT arm (14.7% for ACT vs. 23.0% for CBT, adjusted OR = 0.56 [0.34 to 0.91], *p* = 0.02), but biochemically confirmed PPA rates were not significantly different (9.4% for ACT vs. 13.7% for CBT, adjusted OR = 0.61 [0.34 to 1.11], *p* = 0.10). | 30 days point prevalence abstinence rates at the 12-month follow-up did not differ between  study arms in the primary analysis (13.8% ACT vs. 18.1% CBT, adjusted odds ratio = 0.68  [95% CI = 0.35 to 1.27], *p* = 0.23). |
| Moadel 2012 | USA | RCT | Start of trial: Total: 145, Intervention group: 73, Control group: 72  Analysed: Total: 132, Intervention group: 62, Control group: 70 | HIV positive smokers who are motivated to quit smoking. | 7-day self-reported, point-prevalence abstinence at the final study visit (3 months post quit date). Subjects reporting abstinence with discrepant ECO levels of ≥ 10 parts per million (ppm) were coded as non-abstinent. | The program emphasized the use of a buddy, a supportive friend or family member, to provide encouragement through the quit attempt. Buddies were welcome to attend sessions.  Education about tobacco use and cessation, Role of social and buddy support, Setting quit date. Review of social cues of smoking and strategies to respond to them, Behavioural tools to resist craving, Role of pharmacotherapy. Coping with urges to smoke. | Quit smoking brochure, brief advice to quit (i.e., < five minutes), and an offer of a three-month supply of NRT. | Not measured | At 3 -months - Twenty-one (14.5%) participants achieved the primary cessation outcome, including 14/73 (19.2%) from the treatment group and 7/72 (9.7%) from the control group (O.R.= 1.97 [0.85—4.60], P=0.11) |
| Onyechi 2017 | Nigeria | RCT | Total: 20, Intervention group: 10, Control group: 10 | Prisoners who are current smokers held at Nsukka, Nigeria. | Not stated | Module 1: cognitive restructuring: to help clients correct their problematic thought processes about smoking.  Module 2: mindfulness training: developing attention control, nonjudgmental awareness, and a sense of “true self.”  Module 3: self-modification training: help clients have a direct role in their own treatment. Because this technique aims at self-change, it increases clients’ involvement and commitment to their treatment. Self-modification strategies used include self-monitoring, self-reward, self-contracting, stimulus control, and self-as-model.  Module 4: impulse tolerance and emotional regulation training: help participants develop positive emotions that reflected on their intention to quit smoking. participants were taught how to control impulses and manage their emotions. It includes learning to identify, label, and describe emotions, using mindfulness around the emotional experience, reducing vulnerability to negative emotions.  Module 5: psychodrama: to enable the participants in the study to perceive the health and psychological consequences of smoking, how they could give up smoking habits, and what benefits they could gain by not relapsing. | Standard care | Not measured | There is statistically significant effect of the program on breaking the cigarette-smoking habit among prisoners in the treatment group compared with in the control group: F(1,18)=4.177, P=0.00. |
| Patten 2014 | USA | RCT | Start of trial: Total: 68, Intervention group: 41, Control group: 27  Analysed: Total: 63, Intervention group: 40, Control group: 23 | Inclusion: Age between 12 and 17 years, self-reported smoking in the last 7 days with current tobacco use verified with a salivary cotinine test strip value of >0; willing to make a quit attempt; and had access to a working telephone. | Participants self-reporting no tobacco use in the last 7 days confirmed with a cotinine concentration of ≤15 ng/mL were classified as nontobacco users | The counseling content of the interventions involved efforts to enhance motivation, establish rapport, set goals, promote problem-solving and skill training, and prevent relapse.  Session topics covered reasons for tobacco use, triggers to use tobacco, problem-solving skills  and coping strategies and preventing relapse. Counsellors and teen advisors role-played situations involving social influence, such as when a family member or friend offers the adolescent tobacco. Each adolescent had the opportunity to observe behaviours modelled by others (attention processes) and to enact behaviour (retention processes). Volunteer speakers such as a dentist talked with teens about the health effects of tobacco use. Abstinence from all tobacco products was emphasized as the treatment goal.  To enhance “cultural acceptability,” elders and teen advisors provided intra-treatment support for quitting and shared their personal stories. These individuals shared how tobacco had affected their family and community and why quitting is important. | Standard care | Not measured | The 30-day point-prevalence self-reported tobacco abstinence rates for intervention and control participants were 7% (3/41) and 0% (0/27) at week 6 (*p* = .27); and 10% (4/41) and 0% (0/27) at 6 months (*p* = .15). The 7-day point-prevalence self-reported tobacco abstinence rates for intervention and control participants were 10% (4/41) and 0% (0/27) at both assessments (*p* = .15). Only one adolescent in the intervention condition had biochemically confirmed 7-day point-prevalence abstinence at week 6 and none at 6 months. |
| Ramos 2010 | Spain | **RCT-three arms** | Total: 287: Intensive individual intervention (III)- 81, Intensive group intervention (IGI)- 111, Minimal intervention (MI)-95 | Inclusion: Adult Smokers who were prepared to quit smoking.  Excluded: Aged less than 18 years or with severe mental conditions or terminal illnesses | Continued abstinence at 12 months confirmed through CO-oximetry (CO). | Both the III and the IGI consisted of six visits during which the following were provided: counseling, psychological support and standard follow-up. Counseling and psychological support were based on motivational interview techniques that sought to: (a) reinforce in the smoker the motivation to quit smoking and (b) prevent relapses after smoking cessation. | In all three, pharmacological treatment with nicotine derivatives or bupropion was offered as an option at the physician’s discretion. | Not measured | Continued abstinence at 12 months confirmed through CO was 7.4% in the III, 5.4% in the IGI, and 1% in the MI. No significant differences were noted between III and MI on the one hand, and between IGI and MI on the other [RR 7.04 (0.9-7.2) and RR 5.1 (0.6-41.9), respectively]. No differences were noted between IGI and III [RR 0.7 (0.2-2.2)]. |
| Savant 2013 | India | **RCT-three arms** | Total: 150, control- 47, Individual- 50, Group-53 | Participants who were above 18 years of age and wanted to quit tobacco habit were selected. | Biochemical validation of abstinence was done at 6 months interval using cotinine test only on participants who had reported of quitting. | The protocol followed was of 5 As (Ask, Advice, Assess, Assist and Arrange for follow up) and 5 Rs (Relevance, Risks, Rewards, Roadblocks and Relapse) in the individual and group counseling groups in 5 counseling sessions. | Control group received brief advice for 10-15 minutes.  Individual group received the same as group intervention but to individual level rather than on group. | There was significant difference in the quit rates of the participants in the individual counseling group (ICG) and group counseling group (GCG) when compared at 6 months with the control counseling group (CCG). In the individual counseling group was 6% while in group counseling group it was 7.5% after six months of counseling (p<0.05). | Not measured |
| Stanton 2020 | USA | RCT | Start of trial: Total: 442, Intervention group: 216, Control group: 226  Analysed: Total: 372, Intervention group: 176, Control group: 196 | Adult HIV positive smokers who are motivated to quit smoking.  Excluded: (1) pregnancy, (2) previous participation in the trial, (3) contraindication to nicotine patch use | Self-reported confirmed by exhaled carbon monoxide (ECO) level of <10 parts per million constituted biochemically verified 7-day point-prevalence abstinence for the purpose of this study. | The program emphasized the use of a buddy, a supportive friend or family member, to provide encouragement through the quit attempt. Buddies were welcome to attend sessions.  Education about tobacco use and cessation, Role of social and buddy support, Setting quit date. Review of social cues of smoking and strategies to respond to them, Behavioural tools to resist craving, Role of pharmacotherapy. Coping with urges to smoke. | Standard care | At 6 months, 28 participants in treatment group (13.0%) and 30 control participants (13.3%) achieved the primary study outcome of biochemically confirmed, 7-day point-prevalence abstinence [OR = 0.97 (95% CI = 0.56 to 1.69), P = 1.0]. | At 3 months, 28 participants in treatment group (13.0%) and 15 control participants (6.6%) achieved biochemically confirmed, 7-day point-prevalence abstinence [OR = 2.10 (95% CI = 1.10 to 4.14), P = 0.04]. |
| Swain 2021 | India | RCT | Total: 120, control- 60, Treatment -60 | Participants were male employees who were using tobacco in any form(smoking and smokeless) four or more times a week within the past 6 months were included in the study. | The term abstinence of tobacco use in the study meant participants who had quit tobacco since last 1 month. | Each session included mention of facts related to tobacco, dispelling common myths, explanation of tobacco related burden of disease, and benefits of quitting tobacco.  A second session comprised of sharing experiences of group members in quitting tobacco in the last 5 weeks, withdrawal symptoms and measures to overcome them, coping strategies/ prevention of relapse, and hazards of second-hand smoke. | Self-help material (Booklet) | The 6 months post intervention, in the intervention group, 13(21.7%) of the participants had quit tobacco since last one month and 3(5%) in the control group (relative risk (RR) 4.33 (3.72-4.94). | Not measured |
| Van den Brand 2018 | Netherlands | RCT | Start of trial: Total: 604, control- 285, Treatment -319  Analysed: Total: 603, control- 284, Treatment -319 | tobacco-smoking employees and spouses of employees who were at least 18 years of age. | carbon monoxide-validated continuous abstinence at 12 months. | Participants in the intervention group received vouchers for being abstinent (€50 at the end of the training programme, €50 3 months after completion of the programme, €50 after 6 months, and €200 after 12 months).  Participants from the intervention companies received financial incentives of up to €350 for successful smoking cessation, whereas participants from the control companies did not receive financial incentives. | Weekly 90-min session of smoking cessation group training for 7 weeks at the workplace. | At 6 months, the difference in the proportion of participants who were abstinent between the intervention group 145 (45%) and control group 76 (27%) increased to 19% (OR 2.39, 95% CI 1.62–3.52; p<0.0001) | At 12 months after finishing the smoking cessation programme, the proportion of individuals  abstaining from smoking in the intervention group was significantly higher than that in the control group (131 [41%] of 319 *vs* 75 [26%] of 284; OR 1·93, 95% CI 1.31–2.85, p=0.0009.  Directly after completion of the smoking cessation training programme, 266 (83%) of 319 participants in the intervention group and 216 (76%) of 285 participants in the control group were verified quitters (OR 1.77, 95% CI 1.00–3.12; p=0.050).  At 3 months, the proportion of quitters was 172 (54%) of 319 in the intervention group and 125 (44%) of 285 in the control group (OR 1.55, 95% CI 1.07 to 2.24; p=0.021) |
| **Wagner 2016** | USA | RCT | Total: 400, Intervention group: B- 198, Control group: A-202 | Inclusion: Individuals who smoked three or more cigarettes per day and was 18 years or older. Excluded: participants with acute mental problems. | At 9-month self-reported smoking abstinence verified by clinical and para-clinical measures including ECO, and nicotine withdrawal symptoms. | The behavioural contingency management program included incentives for attending sessions and achieving special goals, such as establishing a quit date or staying quit for one week and one month. Incentives were in the form of $5 gift cards for groceries.  The primary difference between the two interventions was the delivery modality (Group A consisted of individual counseling, and Group B was group counseling). NRT was also offered. | Incentives for attending sessions and achieving special goals, such as establishing a quit date or staying quit for one week and one month. Incentives were in the form of $5 gift cards for groceries. | Not measured | Individual or group intervention; participants in both interventions achieved similar cessation rates of 8.9% and 8.6%, respectively. No statistically significant difference emerged between group assignment and quitting smoking (OR=0.9; 95% CI= 0.4–1.9, p-value= 0.717, as shown in Panel “A”). |
| Zheng 2007 | China | RCT | Start of trial: Total: 225, Intervention group: 118, Control group: 107  Analysed: Total: 217, Intervention group: 116, Control group: 101 | Inclusion: 18 years or older; having smoked more than 100 cigarettes in life time and still smoking when they were recruited; and willing to attend a five-session course and to be followed up for at least 6 months. | A smoking quitter was defined by three parameters. First, the average number of cigarettes smoked during the past week was zero. Second, the subject was in early action or maintenance stage. The last, the cotinine level of urine displayed less than 25 ng/mL. | The first session discussed components of cigarettes and cigarette smoke, smoking associated with cancer and respiratory disease, how to prepare for quitting, and how to make a record of circumstances for each cigarette smoked.  The second session covered information on tobacco associated with coronary heart diseases and stroke, benefits and difficulties of smoking quitting, some misunderstanding on smoking and quitting, the imagining relaxing method, how to make a plan of quitting smoking.  In the third session, discussion focused on the detrimental effect of passive smoking, advantages versus disadvantages of smoking and quitting, gradual muscle relaxation, and some skills and tips in quitting. The fourth session consisted of calculating the expenditure on smoking, making a decision to be a quitter, and writing a farewell letter to cigarettes. In this session, we also invited some successful quitters to share their experience.  The fifth session involved coping strategies in situation of having pressure to smoke, and how to prevent relapse. | Standard care | The 6-month continuous abstinence rate was 28.0% (33/118) and 2.8% (3/107) in the intervention and control group respectively (absolute risk reduction: 25.4%, 95% CI: 15.8-34.9%). AOR 6.42 (2.46-13.28), P-value 0.001. | Not measured |
