# Supplementary material for: What Are the Effective Components of Group-Based Treatment Programs For Smoking Cessation? A Systematic Review and Meta-Analysis
Source: Nicotine Tob Res. 2023 Apr 27;25(9):1525–37. doi: 10.1093/ntr/ntad068 (PMC10439487; doi:10.1093/ntr/ntad068)
Supplement: ntad068_suppl_Supplementary_Material_S3 [file ntad068_suppl_supplementary_material_s3.docx]

**JBI Quality assessment RCT**

Y – Yes; N – No; NA - not applicable; UC - unclear

| **Questions** | **References** | | | | | | | | | | | | | | | | | | |
| --- | --- | --- | --- | --- | --- | --- | --- | --- | --- | --- | --- | --- | --- | --- | --- | --- | --- | --- | --- |
|  | Asfar 2021 | Borglykke 2008 | Caponnetto 2020 | Copeland 2006 | **Gifford 2011** | Hooper 2017 | Kumar 2012 | Little 2020 | McClure 2020 | Moadel 2012 | Onyechi 2017 | Patten 2014 | Ramos 2010 | Savant 2013 | Stanton 2020 | Swain 2021 | Van den Brand 2018 | **Wagner 2016** | Zheng 2007 |
| Was true randomization used for assignment of participants to treatment groups? | Y | UC | Y | UC | Y | UC | Y | UC | UC | UC | Y | UC | Y | UC | Y | UC | Y | UC | Y |
| Was allocation to treatment groups concealed? | Y | UC | UC | UC | UC | UC | N | UC | N | UC | UC | UC | Y | Y | Y | UC | Y | UC | UC |
| Were treatment groups similar at the baseline? | Y | Y | Y | Y | Y | Y | Y | Y | Y | Y | Y | N | Y | UC | Y | UC | UC | Y | N |
| Were participants blind to treatment assignment? | Y | UC | UC | UC | N | UC | N | UC | N | UC | UC | UC | UC | UC | UC | UC | Y | UC | UC |
| Were those delivering treatment blind to treatment assignment? | UC | UC | UC | Y | UC | UC | N | UC | UC | UC | UC | UC | UC | UC | UC | UC | UC | UC | UC |
| Were outcomes assessors blind to treatment assignment? | UC | UC | UC | UC | UC | UC | N | UC | UC | UC | UC | UC | Y | Y | UC | UC | N | UC | UC |
| Were treatment groups treated identically other than the intervention of interest? | Y | Y | Y | Y | Y | Y | Y | Y | Y | Y | Y | Y | Y | Y | Y | Y | Y | Y | Y |
| Was follow up complete and if not, were differences between groups in terms of their follow up adequately described and analysed? | Y | Y | Y | Y | Y | Y | Y | Y | Y | Y | Y | Y | Y | Y | Y | Y | Y | Y | Y |
| Were participants analysed in the groups to which they were randomized? | Y | Y | Y | Y | Y | Y | Y | Y | Y | Y | Y | Y | Y | Y | Y | Y | Y | Y | Y |
| Were outcomes measured in the same way for treatment groups? | Y | Y | Y | Y | Y | Y | Y | Y | Y | Y | Y | Y | Y | Y | Y | Y | Y | Y | Y |
| Were outcomes measured in a reliable way? | Y | Y | Y | Y | Y | Y | Y | Y | Y | Y | Y | Y | Y | Y | Y | Y | Y | Y | Y |
| Was appropriate statistical analysis used? | Y | Y | Y | Y | Y | Y | Y | Y | Y | Y | Y | Y | Y | Y | Y | Y | Y | Y | Y |
| Was the trial design appropriate, and any deviations from the standard RCT design (individual randomization, parallel groups) accounted for in the conduct and analysis of the trial? | Y | Y | Y | Y | Y | Y | Y | Y | Y | Y | Y | Y | Y | N | Y | Y | Y | Y | Y |
| Total score | 11 | 8 | 9 | 9 | 9 | 8 | 9 | 8 | 8 | 8 | 9 | 7 | 10 | 8 | 10 | 7 | 10 | 8 | 8 |
