# Supplementary material for: What Are the Effective Components of Group-Based Treatment Programs For Smoking Cessation? A Systematic Review and Meta-Analysis
Source: Nicotine Tob Res. 2023 Apr 27;25(9):1525–37. doi: 10.1093/ntr/ntad068 (PMC10439487; doi:10.1093/ntr/ntad068)
Supplement: ntad068_suppl_Supplementary_Material_S4 [file ntad068_suppl_supplementary_material_s4.docx]

**Intervention BCTTs/providers/setting/frequency/duration**

| First author and year | Number of BCTTs included in the intervention | BCTTs included in the intervention | Intercoder reliability (kappa scores) | Intervention provider | Setting | Number of group-based session | Frequency | Duration of each session | Mode of intervention delivery | Last follow-up |
| --- | --- | --- | --- | --- | --- | --- | --- | --- | --- | --- |
| 1. Asfar 2021 | 8 | 1. Goal setting (outcome), 2. Problem solving, 3. Avoidance/reducing exposure to cues for the behaviour, 4. Verbal persuasion about capability, 5. Review outcome goal(s), 6. Pharmacological support, 7. Action planning, 8. feedback on behaviour | 0.86 | Health providers with psychology and/or public health qualification | Community setting (workplace) | 1 | Not applicable | Not stated | Face-to-face | 6 months |
| 1. Borglykke 2008 | 5 | 1. Goal setting (outcome), 2. Information about health Consequences, 3. Information about social and environmental consequences, 4. Pharmacological support, 5. Feedback on behaviour, 6. Social support (practical) | 0.71 | Nurses | Health care setting | 5 | Every week | 120 minutes each | Face-to-face | 12 months |
| 1. Caponnetto 2020 | 4 | 1. Problem solving, 2. Behavioural practice/rehearsal, 3. Goal setting (outcome), 4. Verbal persuasion about Capability | 1.0 | Clinical psychologists | Community setting (workplace) | 4 | Baseline, week 12, week 24, and week 52 | 120 minutes each | Face-to-face | 12 months |
| 1. Copeland 2006 | 3 | 1. Reduce prompts/cues, 2. Problem solving, 3. Avoidance/reducing exposure to cues for the behaviour | 0.73 | Clinical psychologists and clinical psychology doctoral students | Health care setting | 6 | Over a 2-week period | Not stated | Face-to-face | 6 months |
| 1. **Gifford 2011** | 7 | 1. Problem solving, 2. Information about emotional consequences, 3. Self-talk, 4. Reduce prompts/cues, 5. Restructuring the social environment, 6. Pharmacological support, 7. Monitoring of behaviour by others without feedback | 0.82 | Master's-level substance abuse therapist and clinical psychology doctoral students | Health care setting | 10 | Every week | Not stated | Face-to-face | 6 months |
| 1. Hooper 2017 | 8 | 1. Restructuring the social environment, 2. Information about health consequences, 3. Goal setting (outcome), 4. Self-talk, 5. Social support (unspecified), 6. Pharmacological support, 7. Problem solving, 8. Behaviour substitution | 0.91 | Doctoral and masters/bachelors-level co-therapy pairs | University-based research clinic | 8 | Over a 4-week period | 90–120 minutes each | Face-to-face | 6 months |
| 1. Kumar 2012 | 3 | 1. Information about health consequences, 2. Problem solving, 3. Information about social and environmental consequences | 0.73 | physician | Community setting | 2 | Over a 5-week period | 30 minutes each | Face-to-face | 2 months |
| 1. Little 2020 | 4 | 1. Information about health consequences, 2. Avoidance/reducing exposure to cues for the behaviour, 3. Reduce prompts/cues, 4. Comparative imagining of future outcomes | 0.85 | Not stated | Community setting (Air force squadron) | 1 | Not applicable | 40 minutes | Face-to-face | 3 months |
| 1. McClure 2020 | 6 | 1. Goal setting (outcome), 2. Reduce prompts/cues, Feedback on behaviour, 3. Action planning, 4. Behavioural practice/rehearsal, 5. Pharmacological support, 6. Avoidance/reducing exposure to cues for the behaviour | 0.91 | master’s level counsellor | Health care setting | 5 | Every week | 90 minutes each | Face-to-face | 12 months |
| 1. Moadel 2012 | 6 | 1. Social support (emotional), 2. Information about health consequences, 3. Goal setting (outcome), 4. Avoidance/reducing exposure to cues for the behaviour, 5. Reduce prompts/cues, 6. Problem solving | 1.0 | Psychologist and peer-facilitator | Health care settings | 8 | Over a 6-week period | Not stated | Face-to-face | 3 months |
| 1. Onyechi 2017 | 7 | 1. Information about health consequences, 2. Self-monitoring of behaviour, 3. Identification of self as role model, 4. Reduce prompts/cues, 5. Avoidance/reducing exposure to cues for the behaviour, 6. Self-reward, 7. self-talk | 0.91 | Researcher | Community setting (prison) | 5 | Every 2 weeks | 40 minutes each | Face-to-face | 2 and half months (10 weeks) |
| 1. Patten 2014 | 8 | 1. Information about health consequences, 2. Information about social and environmental consequences, 3. Goal setting (outcome), 4. Problem solving, 5. Avoidance/reducing exposure to cues for the behaviour, 6. Behavioural practice/rehearsal, 7. Credible source, 8. Demonstration of the behaviour | 0.92 | Tobacco treatment specialists and/or individuals with a behavioural/social science degree | Community setting | 1 | Over 2 days period | Total of 8 hr | Face-to-face | 6 months |
| 1. Ramos 2010 | 4 | 1. Goal setting (outcome), 2. Action planning, Information about health consequences, 3. Problem solving, 4. Avoidance/reducing exposure to cues for the behaviour | 0.85 | physician and nurse | Health care settings | 6 | Not stated | Not stated | Face-to-face | 12 months |
| 1. Savant 2013 | 6 | 1. Information about health consequences, 2. Information about social and environmental consequences, 3. Goal setting (outcome), 4. Problem solving, 5. Avoidance/reducing exposure to cues for the behaviour, 6. Reward (outcome) | 0.88 | Dentists | Community setting (workplace) | 5 | Over a 6-months period | 45 minutes each | Face-to-face | 6 months |
| 1. Stanton 2020 | 7 | 1. Social support (emotional), 2. Information about health consequences, 3. Goal setting (outcome), 4. Avoidance/reducing exposure to cues for the behaviour, 5. Reduce prompts/cues, 6. Problem solving, 7. Pharmacological support | 1.0 | Masters or doctoral training in psychology or social work, and peers | Health care settings | 8 | Over 6-weeks period | Not stated | Face-to-face | 6 months |
| 1. Swain 2021 | 4 | 1. Information about health consequences, 2. Problem solving, 3. Information about social and environmental consequences, 4. Avoidance/reducing exposure to cues for the behaviour | 0.85 | Researcher | Health care settings | 2 | Every 5 weeks | 30 minutes each | Face-to-face | 6 months |
| 1. Van den Brand 2018 | 3 | 1. Incentive (outcome), 2. Information about health consequences, 3. Social support (unspecified) | 1.0 | Professional coaches who are experienced in giving smoking  cessation group training in a workplace setting | Community setting (workplace) | 7 | Every week | 90 minutes | Face-to-face | 12 months |
| 1. **Wagner 2016** | 2 | 1. 1. Material incentive (behaviour), 2. 2. Incentive (outcome) | 1.0 | Physician, nurse, social worker | Health care setting | 12 | Not stated | Not stated | Face-to-face | 9 months |
| 1. Zheng 2007 | 8 | 1. Information about health consequences, 2. Self-monitoring of behaviour, 3. Information about social and environmental consequences, 4. Action planning, 5. Problem solving, 6. Avoidance/reducing exposure to cues for the behaviour, 7. Demonstration of the behaviour, 8. Social reward | 1.0 | Health education professionals | Community setting | 5 | Twice a week | 120 minutes each | Face-to-face | 6 months |
