# Supplementary material for: What Are the Effective Components of Group-Based Treatment Programs For Smoking Cessation? A Systematic Review and Meta-Analysis
Source: Nicotine Tob Res. 2023 Apr 27;25(9):1525–37. doi: 10.1093/ntr/ntad068 (PMC10439487; doi:10.1093/ntr/ntad068)
Supplement: ntad068_suppl_Supplementary_Material_S5 [file ntad068_suppl_supplementary_material_s5.docx]

**Supplementary material: Forest plots, funnel plots and Egger test for the 10 BCT domains**

| BCT Domain | Forest plot | Funnel plot | Regression based Egger test |
| --- | --- | --- | --- |
| 1. Goals and planning |  |  | H0: beta1 = 0; no small-study effects  beta1 = 3.21  SE of beta1 = 1.562  z = 2.05  Prob > \|z\| = 0.0399 |
| 1. Feedback and monitoring |  |  | H0: beta1 = 0; no small-study effects  beta1 = 10.15  SE of beta1 = 2.461  z = 4.12  Prob > \|z\| = 0.0000 |
| 1. Social support |  | **** | H0: beta1 = 0; no small-study effects  beta1 = -5.89  SE of beta1 = 2.279  z = -2.59  Prob > \|z\| = 0.0097 |
| 1. Natural consequences |  |  | H0: beta1 = 0; no small-study effects  beta1 = 2.06  SE of beta1 = 1.505  z = 1.37  Prob > \|z\| = 0.1715 |
| 1. Associations |  | **** | H0: beta1 = 0; no small-study effects  beta1 = -2.04  SE of beta1 = 2.112  z = -0.96  Prob > \|z\| = 0.3354 |
| 1. Repetition and substitution |  | **** | H0: beta1 = 0; no small-study effects  beta1 = 10.40  SE of beta1 = 11.334  z = 0.92  Prob > \|z\| = 0.3590 |
| 1. Reward and threat |  | **** | H0: beta1 = 0; no small-study effects  beta1 = 1.13  SE of beta1 = 1.918  z = 0.59  Prob > \|z\| = 0.5570 |
| 1. Regulation |  |  | H0: beta1 = 0; no small-study effects  beta1 = 2.37  SE of beta1 = 3.691  z = 0.64  Prob > \|z\| = 0.5207 |
| 1. Antecedents |  |  | H0: beta1 = 0; no small-study effects  beta1 = 1.65  SE of beta1 = 1.457  z = 1.13  Prob > \|z\| = 0.2567 |
| 1. Self-belief |  | **** | H0: beta1 = 0; no small-study effects  beta1 = 5.15  SE of beta1 = 4.972  z = 1.04  Prob > \|z\| = 0.3000 |
