# Supplementary material for: What Are the Effective Components of Group-Based Treatment Programs For Smoking Cessation? A Systematic Review and Meta-Analysis
Source: Nicotine Tob Res. 2023 Apr 27;25(9):1525–37. doi: 10.1093/ntr/ntad068 (PMC10439487; doi:10.1093/ntr/ntad068)
Supplement: ntad068_suppl_Supplementary_Material_S6 [file ntad068_suppl_supplementary_material_s6.docx]

**Supplementary material: Forest plots, funnel plots and Egger test for the 15 for BCTs**

| BCTT | Forest plot | Funnel plot | Regression based Egger test |
| --- | --- | --- | --- |
| 1. Goal setting (outcome) |  |  | H0: beta1 = 0; no small-study effects  beta1 = 0.75  SE of beta1 = 2.054  z = 0.36  Prob > \|z\| = 0.7152 |
| 1. Problem solving |  |  | H0: beta1 = 0; no small-study effects  beta1 = 0.41  SE of beta1 = 2.015  z = 0.20  Prob > \|z\| = 0.8386 |
| 1. Action planning |  |  | H0: beta1 = 0; no small-study effects  beta1 = 10.47  SE of beta1 = 2.484  z = 4.22  Prob > \|z\| = 0.0000 |
| 1. Feedback on behaviour |  |  | H0: beta1 = 0; no small-study effects  beta1 = 11.51  SE of beta1 = 13.948  z = 0.83  Prob > \|z\| = 0.4092 |
| 1. Social support (unspecified) |  |  | H0: beta1 = 0; no small-study effects  beta1 = -5.54  SE of beta1 = 9.828  z = -0.56  Prob > \|z\| = 0.5732 |
| 1. Information about health Consequences |  |  | H0: beta1 = 0; no small-study effects  beta1 = 1.44  SE of beta1 = 2.089  z = 0.69  Prob > \|z\| = 0.4899 |
| 1. Information about social and environmental consequences |  |  | H0: beta1 = 0; no small-study effects  beta1 = -18.49  SE of beta1 = 24.861  z = -0.74  Prob > \|z\| = 0.4570 |
| 1. Reduce prompts/cues |  |  | H0: beta1 = 0; no small-study effects  beta1 = -2.04  SE of beta1 = 2.112  z = -0.96  Prob > \|z\| = 0.3354 |
| 1. Behavioural practice/rehearsal |  |  | H0: beta1 = 0; no small-study effects  beta1 = 28.53  SE of beta1 = 28.878  z = 0.99  Prob > \|z\| = 0.3232 |
| 1. Reward (outcome) |  |  | H0: beta1 = 0; no small-study effects  beta1 = -1.00  SE of beta1 = 2.284  z = -0.44  Prob > \|z\| = 0.6631 |
| 1. Pharmacological support |  |  | H0: beta1 = 0; no small-study effects  beta1 = 2.37  SE of beta1 = 3.691  z = 0.64  Prob > \|z\| = 0.5207 |
| 1. Avoidance/reducing exposure to cues for the behaviour |  |  | H0: beta1 = 0; no small-study effects  beta1 = 1.71  SE of beta1 = 2.017  z = 0.85  Prob > \|z\| = 0.3956 |
| 1. Restructuring the social environment |  |  | H0: beta1 = 0; no small-study effects  beta1 = 2.71  SE of beta1 = 4.724  z = 0.57  Prob > \|z\| = 0.5664 |
| 1. Verbal persuasion about capability |  |  | H0: beta1 = 0; no small-study effects  beta1 = -221.65  SE of beta1 = 249.937  z = -0.89  Prob > \|z\| = 0.3752 |
| 1. Self-talk |  |  | H0: beta1 = 0; no small-study effects  beta1 = 2.71  SE of beta1 = 4.724  z = 0.57  Prob > \|z\| = 0.5664 |
